# High-Dose Convalescent Plasma for Treatment of Severe COVID-19 (response)

**DOI:** 10.3201/eid2805.220363

**Published:** 2022-05

**Authors:** Gil C. De Santis, Rodrigo T. Calado

**Affiliations:** University of São Paulo, São Paulo, Brazil

**Keywords:** COVID-19, 2019 novel coronavirus disease, coronavirus disease, severe acute respiratory syndrome coronavirus 2, SARS-CoV-2, viruses, respiratory infections, zoonoses, convalescent plasma, neutralizing antibody, passive immunization, Brazil

**In Response:** We thank Focosi and Casadevall for their comments (*1*). One strong contribution of our study was the high dose (i.e., 1,800 mL in 3 days) of coronavirus disease (COVID-19) convalescent plasma (CCP), which, in our opinion, would be more likely to benefit patients than a lower dose (e.g., 200–600 mL in 1 or 2 doses), as is the protocol in most CCP studies (including but not limited to COVID-19 treatment) (*2*).

The weak point of our study was the relatively large therapeutic window (up to 10 days of signs/symptoms) for CCP transfusion, which may have included the later inflammatory process of illness. One early trial suggested benefit for COVID-19 patients who received CCP within the first 14 days (*3*). Nevertheless, subsequent trials showed that CCP (or serum) administration could be most beneficial for COVID-19 patients when administered as prophylaxis or within the first days of infection (*4*,*5*), ideally, within the first 3 days (*6*) but perhaps not later (*7*,*8*). We emphasize that CCP transfusion was considered experimental at the beginning of the pandemic, and inclusion criteria comprised only patients with severe illness, for whom >7 days of infection are needed for illness to become evident.

We think that applying the suggested formula to identify which COVID-19 patients are likely to benefit from CCP (higher risk for progression to severe disease) would not be applicable to our study because it was envisaged for patients not receiving mechanical ventilation (*9*), whereas the patients in our study had severe disease (90% receiving mechanical ventilation). 

In summary, our study emphasizes that CCP should not be transfused late in the course of disease, when the clinical course is driven by inflammation. This conclusion does not exclude the possibility of transfusing CCP as soon as patients are identified for potential benefit, as suggested by other studies (*6**,**7*).
